# Comparing body temperature measurements using the double sensor method within a wearable device with oral and core body temperature measurements using medical grade thermometers—a short report

**DOI:** 10.3389/fphys.2023.1279314

**Published:** 2023-11-14

**Authors:** Arik Eisenkraft, Nir Goldstein, Meir Fons, Michael Tabi, Anna Danielle Sherman, Arik Ben Ishay, Roei Merin, Dean Nachman

**Affiliations:** ^1^ Institute for Research in Military Medicine, Faculty of Medicine, The Hebrew University of Jerusalem and the Israel Defense Force Medical Corps, Jerusalem, Israel; ^2^ Biobeat Technologies Ltd., Petah Tikva, Israel; ^3^ Heart Institute, Hadassah Medical Center, The Hebrew University of Jerusalem, Jerusalem, Israel

**Keywords:** heat-flux sensor, continuous monitoring, double sensor method, remote patient monitoring, wearable monitoring

## Abstract

**Introduction:** Body temperature is essential for diagnosing, managing, and following multiple medical conditions. There are several methods and devices to measure body temperature, but most do not allow continuous and prolonged measurement of body temperature. Noninvasive skin temperature sensor combined with a heat flux sensor, also known as the “double sensor” technique, is becoming a valuable and simple method for frequently monitoring body temperature.

**Methods:** Body temperature measurements using the “double sensor” method in a wearable monitoring device were compared with oral and core body temperature measurements using medical grade thermometers, analyzing data from two prospective clinical trials of different clinical scenarios. One study included 45 hospitalized COVID-19 patients in which oral measurements were taken using a hand-held device, and the second included 18 post-cardiac surgery patients in which rectal measurements were taken using a rectal probe.

**Results:** In study 1, Bland-Altman analysis showed a bias of −0.04°C [0.34–(−0.43)°C, 95% LOA] with a correlation of 99.4% (*p* < 0.001). In study 2, Bland-Altman analysis showed a bias of 0.0°C [0.27–(−0.28)°C, 95% LOA], and the correlation was 99.3% (*p* < 0.001). In both studies, stratifying patients based on BMI and skin tone showed high accordance in all sub-groups.

**Discussion:** The wearable monitor showed high correlation with oral and core body temperature measurements in different clinical scenarios.

## 1 Introduction

Body temperature is regarded as one of the basic measurements to assess a person’s physiological status. Within the clinical routine, trained medical staff measure and record the patient’s temperature as it is one of the significant components of the diagnosis, management and follow-up of several acute and chronic medical conditions, including suspected infection (NICE, 2016; Khan et al., 2021; Machin et al., 2021). If the temperature is inaccurately measured, a patient may not receive timely treatment, leading to additional morbidity or even mortality. Fever and hypothermia can indicate an increased risk of severe illness and death (NICE, 2016), and incorrect temperature measurement increases the likelihood of less effective clinical interventions (Machin et al., 2021). Currently-used clinical guidelines support decision-making by employing thresholds for each vital sign to define which patients require various interventions, i.e., medical treatment, further investigation, or stepping up of care (Bone et al., 1992; RCP, 2017; PHE, 2020). There are several methods and devices to measure body temperature, but most do not allow continuous and prolonged measurement of body temperature values. Peripheral thermometers estimate body temperature non-invasively at measurement sites such as the ear canal, axilla, skin surface, forehead, temporal artery, and mouth, offering an advantage over invasive devices due to their measurement speed and convenient access to measurement location (Kresch, 1984; Muma et al., 1991; Morley et al., 1992; Wilshaw et al., 1999; Cattaneo et al., 2000; Jean-Mary et al., 2002; Suleman et al., 2002; Callanan, 2003; Crawford et al., 2006; Kimberger et al., 2007; Kimberger et al., 2009). However, in all of these methods, the temperature is assessed through the skin surface, and this can be difficult, as skin temperature is lower than core body temperature and can be influenced by external factors such as peripheral blood perfusion and ambient temperature (Jefferies et al., 2011; Eshraghi et al., 2014). The oral temperature may better express the core body temperature, but it is influenced by various activities such as eating and drinking, and accurate measurement takes longer (Lim et al., 2008). Commercially available noninvasive thermometers include infrared forehead thermometers, infrared tympanic thermometers, temporal artery thermometers, digital sublingual thermometers, heat flux double sensor thermometers, and thermal imaging cameras (Mah et al., 2021). A noninvasive skin temperature sensor combined with a heat flux sensor, also known as the “double sensor” technique, is gaining more traction as a useful and simple method for frequently monitoring body temperature (Gunga et al., 2009; Janke et al., 2021). Heat flux sensors provide a quicker response time and increased wearing comfort while delivering comparable results to measurements taken at the same time using other devices (Gunga et al., 2009; Kimberger et al., 2009; Kimberger et al., 2013). As a digital tool and remote patient monitoring gain traction, there is a need for noninvasive and wireless systems that will allow continuous body temperature monitoring, which will help health systems to implement future strategies with dramatic changes in the way clinical data is collected. This will also allow improved monitoring of subjects at home, improving home hospitalization, pre-hospital care, and post-hospital care and providing better tools for prevention and early detection and alert of different medical emergencies, especially infectious diseases (Eisenkraft et al., 2023). The aim of the current study was to compare the body temperature measurements obtained by a wearable wireless remote patient monitoring (RPM) device using the “double sensor” method with oral and core body temperature measurements obtained using medical-grade thermometers in two critical clinical scenarios, as required by regulatory agencies.

## 2 Materials and methods

### 2.1 Ethical consideration

This study includes data from two prospective, comparative clinical trials approved by the Institutional Review Board of the Baruch Padeh Medical Center, Poriya, Israel (study 1: 0077-18-POR NCT03603860 and study 2: 0048-20-POR). All participants were advised both orally and in writing about the nature of the clinical study and signed an informed consent form.

### 2.2 Study outline

The wearable “double sensor” based thermometer was compared to acceptable medical grade thermometers in two clinical scenarios - patients with acute viral respiratory disease and post-surgical ventilated patients. Measurements were taken when all patients were at rest, in bed.

Study 1: 45 COVID-19 patients (ages 18-96; 29 males) admitted to an isolation unit were recruited for the study. Monitoring started immediately after admission. Temperature measurements were compared between the wearable chest patch and oral measurements taken using a hand-held SureTemp Plus 690 (Welch Allyn, Auburn, NY, United States) thermometer.

Study 2: Eighteen post-cardiac surgery patients (ages 18–81 years; 12 males) were recruited for the study. Upon arrival at the Cardiac Surgery Intensive Care Unit (CSICU), monitoring started immediately after the surgical procedure. Upon arrival at the CICU they were connected to an MP60 IntelliVue Patient Monitor (Koninklijke Philips N.V, Amsterdam, Netherlands), and in parallel, they received the wearable chest patch monitor. All parameters derived from the hospital monitoring systems were recorded once every minute using the medical center’s electronic medical record system, and data from the wearable devices were automatically collected and recorded in the company’s cloud. Core temperature measurements were taken rectally using the thermometer of the MP60 IntelliVue Patient Monitor, inserted to a depth of no less than 15 cm into the rectum.

In both studies, temperatures of 34°C–42°C were included. The inclusion criteria were male and female patients 18 years and older. Exclusion criteria were pregnancy and patients younger than 18 years.

### 2.3 The wearable device

**FIGURE 1:**
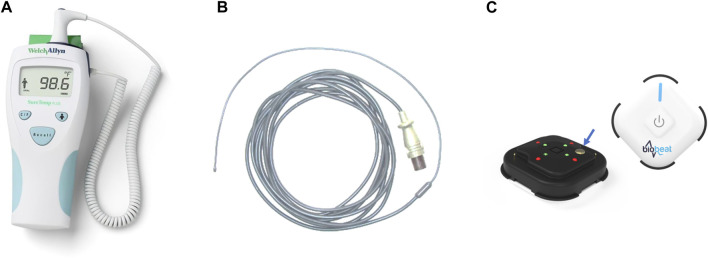
The devices used in the studies to measure the temperature. **(A)** a hand-held SureTemp Plus 690 (Welch Allyn, Auburn, NY, United States) oral thermometer; **(B)** a rectal temperature probe, connected to an MP60 IntelliVue Patient Monitor (Koninklijke Philips N.V, Amsterdam, Netherlands); **(C)** BB-613P, Biobeat Technologies Ltd., Petach Tikva, Israel. Front and rear view of the sensor are presented. The blue arrow shows the location of the thermistor measuring the patient’s temperature.

The wearable chest patch device (BB-613P, Biobeat Technologies Ltd., Petah Tikva, Israel) is a wireless, wearable, noninvasive device that implements reflective photoplethysmography (PPG) technology which allows capturing unique characteristics of the PPG wave, including original wave markers. The PPG sensor provides pulse rate, respiratory rate, cuffless blood pressure, blood oxygen saturation, stroke volume, cardiac output, and more (Eisenkraft et al., 2023). On top of the PPG sensor, the chest patch provides body temperature using the double sensor method (Figure 1), in which body temperature is calculated from two temperature thermistors separated by an insulating disk with a known heat transfer coefficient. Various studies have suggested the reliability and validity of heat-flux approaches and double sensor technology under different environmental conditions, as well as in clinical conditions (Sakuragi et al., 1993; Gunga et al., 2009; Kimberger et al., 2009; 2013; Teunissen et al., 2011; Xu et al., 2013; Mazgaoker et al., 2017; Mendt et al., 2017; Gómez-Romero et al., 2019; Janke et al., 2021; Masè et al., 2022). Integrating this sensor allows for the first time the collection of all five basic vital signs needed in clinical practice using a single wearable device.

### 2.4 Statistical analysis

Correlation analysis was performed using Pearson’s correlation, and agreement was evaluated based on the Bland-Altman method using 95% limits of agreement (LOA). Patients were stratified by BMI and skin tone based on the Fitzpatrick scale (Fitzpatrick, 1988). Analyses were performed by using IBM SPSS Statistics for Windows, version 25.0, IBM Corp (Armonk, NY). The analysis team had no access to the clinical data of the participants.

## 3 Results

Supplementary Table S1 include demographic data and characteristics of the participants in both studies. No adverse events were recorded in any of the participants in both studies.

Study 1 included 45 samples, one from each patient. 18 (40%) were from patients with normal weight (BMI <25), 15 (33%) were from patients with overweight (25 ≤ BMI <30), 12 (27%) were from patients with obesity (30 ≤ BMI), 32 (71%) were from patients with Fitzpatrick 1-3, and 13 (29%) were from patients with Fitzpatrick 4-6.

**FIGURE 2:**
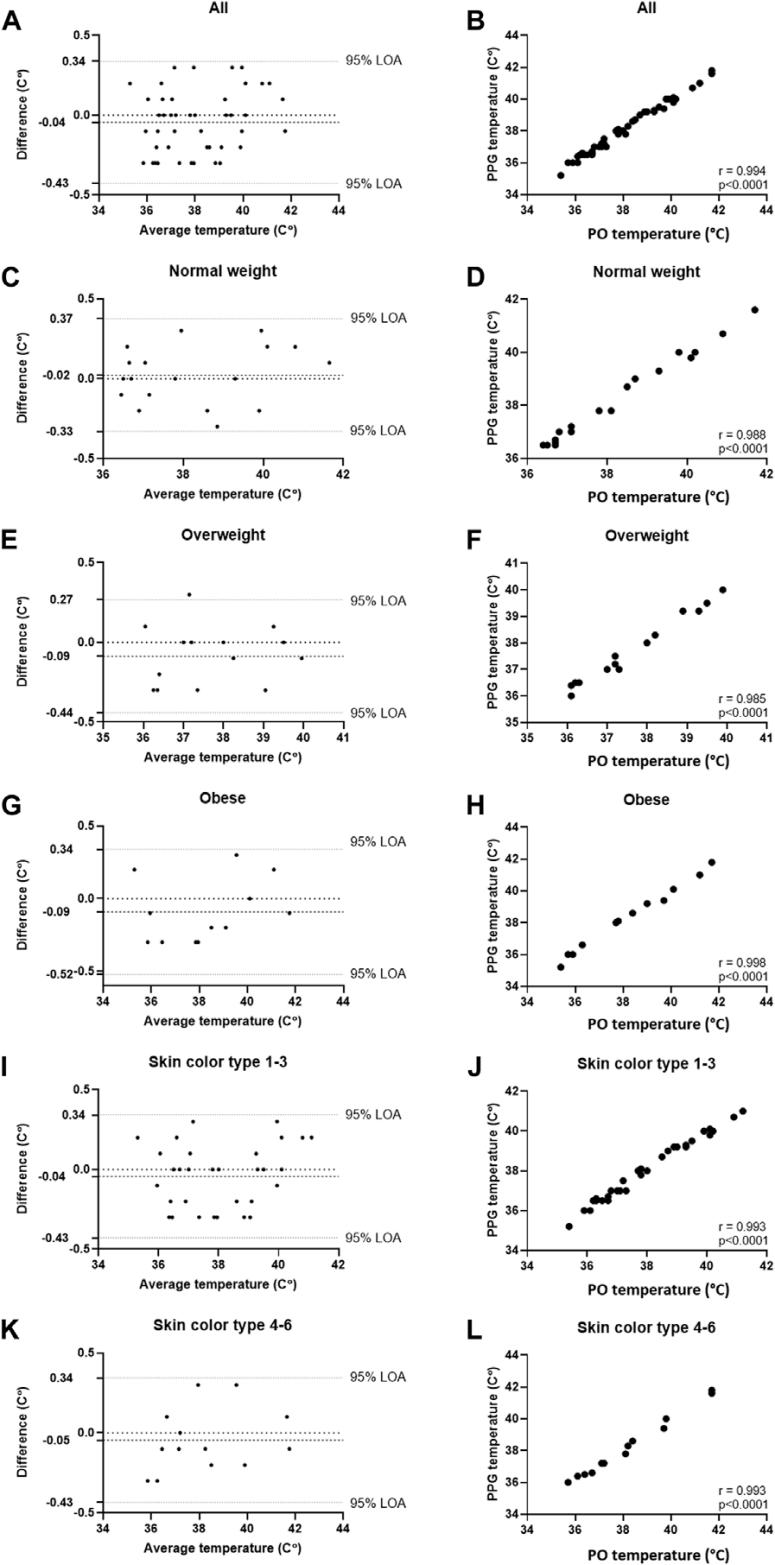
Comparing temperature measurements between the wearable PPG-based device and the handheld SureTemp Plus 690 (Welch Allyn, Auburn, NY, United States) oral thermometer in 45 COVID-19 patients (45 samples). **(A)** Bland-Altman analysis of all patients; **(B)** Pearson’s correlation curve of all patients; **(C)** Bland-Altman analysis of patients with normal weight (BMI <25); **(D)** Pearson’s correlation curve of patients with normal weight (BMI <25); **(E)** Bland-Altman analysis of patients with overweight (25 ≤ BMI ≤30); **(F)** Pearson’s correlation curve of patients with overweight (25 ≤ BMI ≤30); **(G)** Bland-Altman analysis of patients with obesity (30 ≤ BMI); **(H)** Pearson’s correlation curve of patients with obesity (30 ≤ BMI); **(I)** Bland-Altman analysis of patients with Fitzpatrick 1-3; **(J)** Pearson’s correlation curve of patients with Fitzpatrick 1-3; **(K)** Bland-Altman analysis of patients with Fitzpatrick 4-6; **(L)** Pearson’s correlation curve of patients with Fitzpatrick 4-6.

**FIGURE 3:**
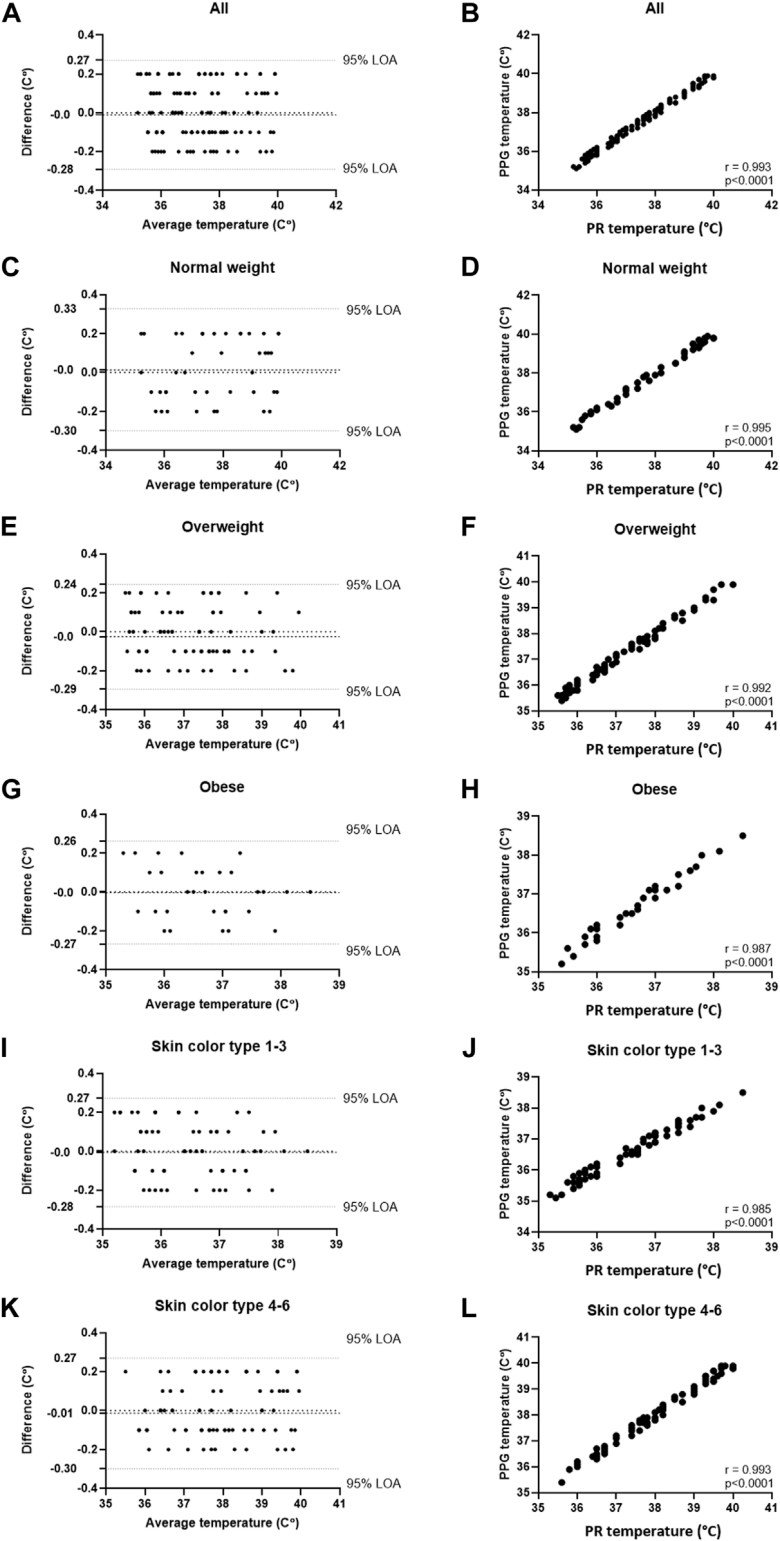
Comparing temperature measurements between the wearable PPG-based device and a rectal probe connected to an MP60 IntelliVue Patient Monitor (Koninklijke Philips N.V, Amsterdam, Netherlands) in 18 post-cardiac surgery patients (161 samples). **(A)** Bland-Altman analysis of all patients; **(B)** Pearson’s correlation curve of all patients; **(C)** Bland-Altman analysis of patients with normal weight (BMI <25); **(D)** Pearson’s correlation curve of patients with normal weight (BMI <25); **(E)** Bland-Altman analysis of patients with overweight (25 ≤ BMI ≤30); **(F)** Pearson’s correlation curve of patients with overweight (25 ≤ BMI ≤30); **(G)** Bland-Altman analysis of patients with obesity (30 ≤ BMI); **(H)** Pearson’s correlation curve of patients with obesity (30 ≤ BMI); **(I)** Bland-Altman analysis of patients with Fitzpatrick 1-3; **(J)** Pearson’s correlation curve of patients with Fitzpatrick 1-3; **(K)** Bland-Altman analysis of patients with Fitzpatrick 4-6; **(L)** Pearson’s correlation curve of patients with Fitzpatrick 4-6.

Table 1 details the results of the comparisons in both studies, including Bland-Altman analysis (Bias and 95% LOA) and correlations (% and *p*). High correlation was found in both studies (r = 0.994 and 0.993, respectively, *p* < 0.001 for both). In Study 1, Bland-Altman analysis of all participants showed a bias of −0.04°C 0.34 – (−0.43) °C, 95% LOA, and in Study 2, Bland-Altman analysis of all participants showed a bias of 0.0°C 0.27 – (−0.28) °C, 95% LOA. In all, high accordance was found in all sub-groups. The detailed Bland-Altman and correlation curves are also included, with data from Study 1 provided in Figure 2, and data from Study 2 provided in Figure 3.

**TABLE 1:** Bland-Altman analysis and correlation analysis of the two studies.

	Study 1	Study 2
Bland-Altman analysis of samples from all participants, Bias (95% LOA), °C	−0.04 (0.34 – (−0.43))	0.0 (0.27 – (−0.28))
Correlation of samples from all participants, % (*p*)	99.4% (*p* < 0.001)	99.3% (*p* < 0.001)
Bland-Altman analysis of samples from normal weight participants, Bias (95% LOA), °C	−0.02 (0.37 – (−0.33))	0.0 (0.33 – (−0.30))
Correlation of samples from normal weight participants, % (*p*)	98.8% (*p* < 0.001)	99.5% (*p* < 0.001)
Bland-Altman analysis of samples from participants with overweight, Bias (95% LOA), °C	−0.09 (0.27 – (−0.44))	0.0 (0.24 – (−0.29))
Correlation of samples from participants with overweight, % (*p*)	98.5% (*p* < 0.001)	99.2% (*p* < 0.001)
Bland-Altman analysis of samples from participants with obesity, Bias (95% LOA), °C	−0.09 (0.34 – (−0.52))	0.0 (0.26 – (−0.27))
Correlation of samples from participants with obesity, % (*p*)	99.8% (*p* < 0.001)	98.7% (*p* < 0.001)
Bland-Altman analysis of samples from participants with Fitzpatrick 1-3, Bias (95% LOA), °C	−0.04 (0.34 – (−0.43))	0.0 (0.27 – (−0.28))
Correlation of samples from participants with Fitzpatrick 1-3, % (*p*)	99.3% (*p* < 0.001)	98.5% (*p* < 0.001)
Bland-Altman analysis of samples from participants with Fitzpatrick 4-6, Bias (95% LOA), °C	−0.05 (0.34 – (−0.43))	−0.01 (0.27 – (−0.30))
Correlation of samples participants with Fitzpatrick 4-6, % (*p*)	99.3% (*p* < 0.001)	99.3% (*p* < 0.001)

Study 1 included 45 samples, one from each participant. Study 2 included 161 samples from 18 participant. Stratification based on Body Mass Index (BMI) considered BMI <25 as normal weight, 25 ≤ BMI <30 as participants with overweight, and 30 ≤ BMI as participants with obesity. Skin tone stratification included Fitzpatrick 1-3 and Fitzpatrick 4-6. LOA - limits of agreement. Significance was considered as *p* < 0.05.

## 4 Discussion

In this study we show that the double sensor method integrated within a wearable chest patch sensor provides accurate and valid temperature measurements in a diverse population of patients.

In most clinical settings, temperature is collected as a single snap shot in time. This, together with frequent lack of contextual information on the clinical condition or circadian variabilities from baseline at the time of measurement, makes the assessment of fever from a single-thermometry assessment reliable only when the fever is well outside the range of normal variations (Smarr et al., 2020). Moreover, current reliance on single-point temperature assessment can lead to missed case identification, as was demonstrated in the COVID-19 pandemic, in which the rates showed no decline despite growing use of temperature checks in multiple places. Essentially, the question is if continuous temperature observations can allow us clinical and medical insights that we might miss when using single-point measurements. We think that continuous or frequent temperature measurement using wearable devices could provide important contextual information, rendering temperature data and fever detection more useful in multiple clinical conditions, especially important among oncology patients with low white blood cell counts, elderly suffering from infection such as pneumonia, and across whole populations in the context of pandemics (Abbasi, 2017; Smarr et al., 2020). As the currently-studied device measures multiple physiological parameters, including temperature, looking at changes of several variables continuously and at the same time could help improve our understanding of the clinical status of the monitored individual. This is true especially in cases of infection, in which inflammatory responses lead to changes in several physiological parameters, and could allow better illness prediction and early detection even before clinical signs and symptoms are evident (Smarr et al., 2020; Goldstein et al., 2021). Assuming these profiles systematically vary by medical condition, they make time series data of temperature, blood pressure, heart rate, respiratory rate and more, useful as early-stage indicators in illness detection and determination, and could help capture the dynamic nature of a given medical condition more effectively than temperature alone. Future studies should further compare measurements from such wearable devices with core body temperature measurements, to further elucidate whether these devices could replace the more cumbersome rectal thermistors, allowing for accurate temperature measurements in multiple clinical settings.

A simple and accurate temperature measurement collected using a sensor that also provides numerous other vital signs could be of clinical importance when aiming to help with the workflow of nursing staff in various clinical settings. The wearable sensor tested in this study provides multiple physiological parameters, including the five basic parameters collected in clinical practice - cuffless blood pressure, pulse rate, respiratory rate, blood oxygen saturation, and temperature. The role of the combined measured parameters is under research, looking at potential future directions in the clinical management of various conditions (Eisenkraft et al., 2023). Though the thermistors are not expected to measure the temperature differently in people with various skin tones and BMI, this was still a request by regulatory agencies, to assure no apparent differences between various populations.

As mentioned earlier, the rectal thermometer was inserted into a depth of no less than 15 cm, known to be the most valid depth of a flexible rectal thermistor (Miller et al., 2017). All patients were in bed and at rest during the study.

Combining all these parameters in a single wearable and wireless device, which transmits the data automatically to a management platform integrated into various EMR systems would transform the way clinical practice is conducted, as it will facilitate proper monitoring, early recognition of deterioration, and even more importantly, will enable early intervention based on risk score (Eisenkraft et al., 2023).

A limitation of this study is the relatively small number of patients in each group, however, despite this limitation, we manage to show high accordance between the measurement methods. Future studies will look at how continuous monitoring of body temperature, combined with other physiological parameters, could help with decisions regarding admission and discharge from hospital, and with long-term follow up of patients wherever they are, either out-of-hospital, at home, or in the hospital.

To conclude, the temperature recordings provided by the wearable chest patch used in this study show high correlation and accordance with temperature measurements recorded with oral and rectal medical grade thermometers.

## Data Availability

The original contributions presented in the study are included in the article/Supplementary Material, further inquiries can be directed to the corresponding author.
