# Supplementary material for: Comparing body temperature measurements using the double sensor method within a wearable device with oral and core body temperature measurements using medical grade thermometers—a short report
Source: Front Physiol. 2023 Nov 14;14:1279314. doi: 10.3389/fphys.2023.1279314 (PMC10685445; doi:10.3389/fphys.2023.1279314)
Supplement: Supplementary file 1 [file Table1.DOCX]

**Comparing Body Temperature Measurements Using the Double Sensor Method Within a Wearable Device with Oral and Core Body Temperature Measurements Using Medical Grade Thermometers – a Short Report**

**Supplemental Table 1. General demographic data of the participants in both studies.**

| **Gender** | **Age** | **BMI** | **Fitzpatrick Scale** |
| --- | --- | --- | --- |
| **Study 1** | | | |
| M | 28 | 21 | Type ll |
| M | 65 | 29 | Type l |
| M | 38 | 26 | Type lll |
| M | 60 | 30 | Type lll |
| F | 31 | 23 | Type lV |
| M | 96 | 28 | Type ll |
| M | 65 | 25 | Type V |
| M | 36 | 28 | Type l |
| F | 77 | 29 | Type ll |
| F | 94 | 27 | Type lll |
| F | 63 | 21 | Type lV |
| M | 74 | 31 | Type V |
| M | 68 | 26 | Type ll |
| M | 66 | 36 | Type l |
| M | 65 | 29 | Type ll |
| M | 82 | 36 | Type l |
| M | 35 | 22 | Type Vl |
| F | 48 | 25 | Type V |
| M | 49 | 33 | Type l |
| M | 61 | 28 | Type lll |
| F | 79 | 23 | Type lll |
| M | 42 | 31 | Type lV |
| F | 65 | 31 | Type ll |
| F | 65 | 28 | Type V |
| F | 54 | 29 | Type l |
| M | 73 | 21 | Type ll |
| M | 76 | 22 | Type lll |
| M | 41 | 28 | Type lV |
| M | 64 | 26 | Type V |
| M | 51 | 25 | Type ll |
| F | 66 | 24 | Type l |
| F | 57 | 38 | Type ll |
| F | 70 | 22 | Type l |
| M | 60 | 35 | Type l |
| F | 31 | 20 | Type ll |
| M | 90 | 28 | Type lll |
| M | 24 | 25 | Type lV |
| M | 25 | 31 | Type V |
| F | 18 | 22 | Type ll |
| M | 30 | 25 | Type l |
| F | 52 | 23 | Type ll |
| F | 55 | 31 | Type l |
| M | 89 | 31 | Type lll |
| M | 67 | 24 | Type lll |
| M | 62 | 32 | Type lV |
| **Study 2** | | | |
| M | 65 | 33 | Type ll |
| M | 60 | 33 | Type l |
| F | 75 | 25 | Type lll |
| F | 45 | 25 | Type lll |
| F | 45 | 29 | Type lV |
| F | 78 | 25 | Type ll |
| M | 60 | 28 | Type V |
| M | 18 | 42 | Type l |
| M | 81 | 28 | Type ll |
| M | 35 | 26 | Type lll |
| F | 76 | 28 | Type lV |
| M | 64 | 26 | Type V |
| M | 57 | 24 | Type ll |
| M | 71 | 24 | Type l |
| M | 58 | 20 | Type ll |
| M | 70 | 29 | Type l |
| F | 53 | 22 | Type Vl |
| M | 47 | 26 | Type V |
